# Bis(1,10-phenanthroline-κ^2^ *N*,*N*′)bis­(thio­cyanato-κ*N*)cadmium

**DOI:** 10.1107/S160053681101289X

**Published:** 2011-05-07

**Authors:** Daniel Vallejo, Garikoitz Beobide, Oscar Castillo, Antonio Luque

**Affiliations:** a Departamento de Química Inorgánica, Facultad de Ciencia y Tecnología, Universidad del País Vasco, Apdo. 644, E-48080 Bilbao, Spain

## Abstract

The title compound, [Cd(NCS)_2_(C_12_H_8_N_2_)_2_], has been obtained from the decomposition reaction of dithio­oxamide in a dimethyl­formamide solution containing 1,10-phenanthroline (phen) and Cd(NO_3_)_2_·4H_2_O. Its crystal structure is formed by monuclear Cd^II^ entities in which the metal atom is sited on a twofold rotation axis. The Cd^II^ atom is six-coordinated in the form of a distorted octa­hedron by two chelating phenanthroline mol­ecules and two thio­cyanate anions coordinated through their N atoms. In the crystal, C—H⋯N hydrogen bonds are established between the phenanthroline and thio­cyanate ligands of neighbouring complexes.

## Related literature

For the coordination versatility of the thio­cyanate anion in transition metal complexes, see: Goher *et al.* (2000 ▶). For isotypic Mn(II), Fe(II), Co(II), Cu(II) and Zn(II) structures, see: Holleman *et al.* (1994 ▶); Gallois *et al.* (1990 ▶); Yin (2007 ▶); Parker *et al.* (1996 ▶); Liu *et al.* (2005 ▶). For another Cd^II^–phen complex with a CdN_6_ coordination environment, see: He *et al.* (2004 ▶). For Cd—N bond lengths in related structures, see: Moon *et al.* (2000 ▶).
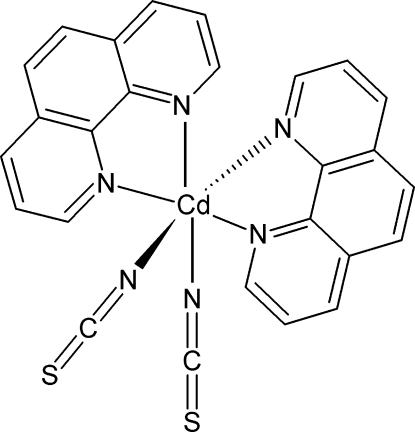


## Experimental

### 

#### Crystal data

[Cd(NCS)_2_(C_12_H_8_N_2_)_2_]
*M* *_r_* = 588.97
Orthorhombic, 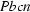

*a* = 13.5295 (2) Å
*b* = 9.91538 (18) Å
*c* = 17.5297 (2) Å
*V* = 2351.62 (6) Å^3^
*Z* = 4
Mo *K*α radiation
μ = 1.14 mm^−1^
*T* = 100 K
0.32 × 0.22 × 0.21 mm

#### Data collection

Oxford Diffraction Xcalibur diffractometer
Absorption correction: analytical (*CrysAlis RED*; Oxford Diffraction, 2003 ▶) *T*_min_ = 0.757, *T*_max_ = 0.826
19593 measured reflections
3444 independent reflections
2606 reflections with *I* > 2σ(*I*)
*R*_int_ = 0.031

#### Refinement

*R*[*F* ^2^ > 2σ(*F* ^2^)] = 0.040
*wR*(*F* ^2^) = 0.117
*S* = 1.11
3444 reflections
159 parameters
H-atom parameters constrained
Δρ_max_ = 1.56 e Å^−3^
Δρ_min_ = −0.83 e Å^−3^

### 

Data collection: *CrysAlis CCD* (Oxford Diffraction, 2003 ▶); cell refinement: *CrysAlis CCD*; data reduction: *CrysAlis RED* (Oxford Diffraction, 2003 ▶); program(s) used to solve structure: *SIR92* (Altomare *et al.*, 1993 ▶); program(s) used to refine structure: *SHELXL97* (Sheldrick, 2008) ▶; molecular graphics: *ORTEP-3* (Farrugia, 1997 ▶); software used to prepare material for publication: *WinGX* (Farrugia, 1999 ▶).

## Supplementary Material

Crystal structure: contains datablocks I, global. DOI: 10.1107/S160053681101289X/zj2006sup1.cif

Structure factors: contains datablocks I. DOI: 10.1107/S160053681101289X/zj2006Isup2.hkl

Additional supplementary materials:  crystallographic information; 3D view; checkCIF report

## Figures and Tables

**Table:** 

Cd—N3	2.262 (3)
Cd—N2	2.369 (3)
Cd—N1	2.372 (3)

**Table:** 

N3—Cd—N3^i^	95.97 (15)
N3—Cd—N2^i^	108.26 (9)
N3—Cd—N2	89.42 (10)
N2^i^—Cd—N2	153.81 (12)
N3—Cd—N1^i^	90.14 (10)
N2—Cd—N1^i^	90.48 (8)
N3—Cd—N1	160.29 (9)
N2—Cd—N1	70.87 (8)
N1^i^—Cd—N1	90.34 (13)

Symmetry code: (i) 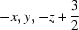.

**Table 2:** Hydrogen-bond geometry (Å, °)

*D*—H⋯*A*	*D*—H	H⋯*A*	*D*⋯*A*	*D*—H⋯*A*
C8—H8⋯N3^ii^	0.93	2.54	3.373 (4)	149

Symmetry code: (ii) 
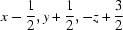
.

**Fig. 1.**
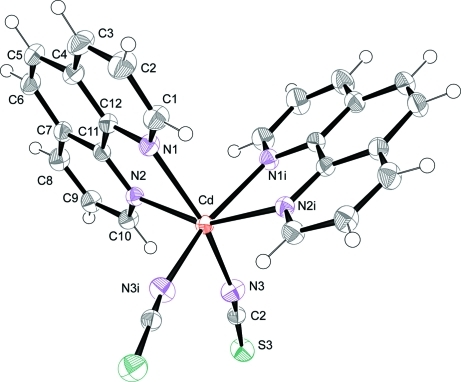
The molecular structure of (I) showing atom labels and 50% probability displacement ellipsoids for non-H atoms. Atoms with sufix i are generated by the symmetry operator (–x, y, 3/2 – z).

**Fig. 2.**
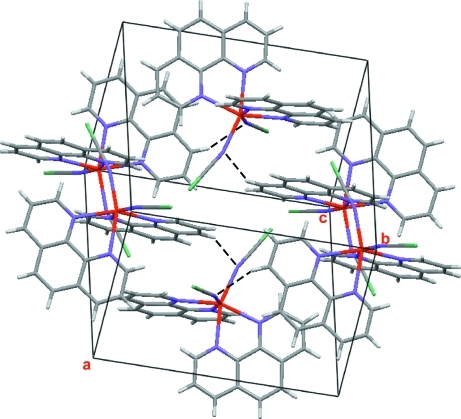
View of the crystal packing of (I) showing the hydrogen bonding scheme.

## supplementary crystallographic information

### Comment

Transition metal complexes based on thiocyanate anion have been widely studied due the coordination versatility of this ligand (Goher *et al.*, 2000). Regarding to the title compound, it deserves to note that isostructural compounds of Mn(II), Fe(II), Co(II), Cu(II) and Zn(II) have been previously reported (Holleman *et al.*, 1994; Gallois *et al.*, 1990; Yin, 2007;Parker *et al.*, 1996; Liu *et al.*, 2005). However, to the best of our knowledge,the crystal structure described herein represents the first example of the Cd^II^ analogue (*I*). The cadmium(II) cation is placed on a twofold rotation axis showing a distorted octahedral coordination geometry. The coordination environment is completed by four N atoms of two chelating phen ligands in *cis* arrangement and by two N atoms of two thiocyanate anions (Fig. 1). The two phen ligands are almost perpendicular to each other, with a dihedral angle of 84.8 (1)°. The Cd—N distances corresponding to chelating phen ligands (*ca* 2.37 Å) are comparable to values found in other Cd^II^-phen complex with CdN_6_ coordination environment (He *et al.*, 2004). While the bond Cd—N distance (2.262 (3) Å) corresponding to thiocyanate N atoms is sligthly shorter and similar to those found in related compounds (Moon *et al.*, 2000). In the crystal structure, neighbouring complexes interact by means of C—H···N hydrogen bondings (Table 2). Figure 2 shows a view of the crystal packing with the hydrogen bonding interaction scheme.

### Experimental

Cd(NO_3_)_2_^.^4H_2_O (43.3 mg, 0.140 mmol), phen (66.4 mg, 0.368 mmol) and dithiooxamide (18.4 mg, 0.153 mmol) were mixed in 30 ml of dimethylformamide. The reaction mixture was stirred for 30 min and subsequently it was allowed to stand in air. Rombohedral yellow crystals were obtained three weeks later. They were filtered out, washed with ethanol and dried at room temperature (yield 40%). Elemental analysis calculated for C_26_H_16_CdN_6_S_2_: C 53.02, H 2.74, Cd 19.08, N 14.27, S 10.89%; found: C 53.96, H 3.01, Cd 18.75, N 13.87, S 10.63%.

### Refinement

H atoms were included at geometrically calculated positions and refined as riding atoms [C—H = 0.93 Å and *U*_iso_(H) = 1.2*U*_eq_(C)].

### Crystal data

**Table:**

[Cd(NCS)_2_(C_12_H_8_N_2_)_2_]	*F*(000) = 1176
*M**_r_* = 588.97	*D*_x_ = 1.664 Mg m^−^^3^
Orthorhombic, *P**b**c**n*	Mo *K*α radiation, λ = 0.71073 Å
Hall symbol: -P 2n 2ab	Cell parameters from 19593 reflections
*a* = 13.5295 (2) Å	θ = 3.0–30.1°
*b* = 9.91538 (18) Å	µ = 1.14 mm^−^^1^
*c* = 17.5297 (2) Å	*T* = 100 K
*V* = 2351.62 (6) Å^3^	Rhombohedral, yellow
*Z* = 4	0.32 × 0.22 × 0.21 mm

### Data collection

**Table:**

Oxford Diffraction Xcalibur diffractometer	3444 independent reflections
Radiation source: fine-focus sealed tube	2606 reflections with *I* > 2σ(*I*)
graphite	*R*_int_ = 0.031
ω scans	θ_max_ = 30.1°, θ_min_ = 3.0°
Absorption correction: analytical (*CrysAlis RED*; Oxford Diffraction, 2003)	*h* = −18→19
*T*_min_ = 0.757, *T*_max_ = 0.826	*k* = −13→10
19593 measured reflections	*l* = −24→23

### Refinement

**Table:**

Refinement on *F*^2^	Primary atom site location: structure-invariant direct methods
Least-squares matrix: full	Secondary atom site location: difference Fourier map
*R*[*F*^2^ > 2σ(*F*^2^)] = 0.040	Hydrogen site location: inferred from neighbouring sites
*wR*(*F*^2^) = 0.117	H-atom parameters constrained
*S* = 1.11	*w* = 1/[σ^2^(*F*_o_^2^) + (0.0606*P*)^2^ + 3.0087*P*] where *P* = (*F*_o_^2^ + 2*F*_c_^2^)/3
3444 reflections	(Δ/σ)_max_ < 0.001
159 parameters	Δρ_max_ = 1.56 e Å^−^^3^
0 restraints	Δρ_min_ = −0.83 e Å^−^^3^

### Special details

**Table:**

Experimental. CrysAlis RED, Oxford Diffraction Ltd., Version 1.170.32 (release 06.06.2003 CrysAlis170 VC++)(compiled Jun 6 2003,13:53:32). Analytical numeric absorption correction using a multifaceted crystal model based on expressions derived by R.C. Clark & J.S. Reid.
Geometry. All e.s.d.'s (except the e.s.d. in the dihedral angle between two l.s. planes) are estimated using the full covariance matrix. The cell e.s.d.'s are taken into account individually in the estimation of e.s.d.'s in distances, angles and torsion angles; correlations between e.s.d.'s in cell parameters are only used when they are defined by crystal symmetry. An approximate (isotropic) treatment of cell e.s.d.'s is used for estimating e.s.d.'s involving l.s. planes.
Refinement. Refinement of *F*^2^ against ALL reflections. The weighted *R*-factor *wR* and goodness of fit *S* are based on *F*^2^, conventional *R*-factors *R* are based on *F*, with *F* set to zero for negative *F*^2^. The threshold expression of *F*^2^ > σ(*F*^2^) is used only for calculating *R*-factors(gt) *etc*. and is not relevant to the choice of reflections for refinement. *R*-factors based on *F*^2^ are statistically about twice as large as those based on *F*, and *R*- factors based on ALL data will be even larger.

### Fractional atomic coordinates and isotropic or equivalent isotropic displacement parameters (Å^2^)

**Table:**

	*x*	*y*	*z*	*U*_iso_*/*U*_eq_	
Cd	0.0000	0.31999 (3)	0.7500	0.01949 (10)	
S1	−0.16408 (7)	0.01037 (9)	0.56558 (5)	0.0346 (2)	
N1	−0.00666 (17)	0.4887 (3)	0.84583 (15)	0.0237 (5)	
N2	−0.16737 (19)	0.3741 (3)	0.77514 (14)	0.0213 (5)	
N3	−0.0504 (2)	0.1673 (3)	0.66235 (16)	0.0308 (6)	
C13	−0.0980 (2)	0.1038 (3)	0.62292 (16)	0.0224 (5)	
C12	−0.0987 (2)	0.5344 (3)	0.86435 (16)	0.0226 (6)	
C9	−0.3426 (2)	0.3557 (4)	0.75684 (17)	0.0283 (6)	
H9	−0.3950	0.3137	0.7321	0.034*	
C11	−0.1831 (2)	0.4731 (3)	0.82731 (15)	0.0215 (5)	
C7	−0.2791 (2)	0.5182 (3)	0.84580 (16)	0.0254 (6)	
C8	−0.3593 (2)	0.4548 (4)	0.80914 (18)	0.0303 (7)	
H8	−0.4236	0.4806	0.8206	0.036*	
C1	0.0703 (3)	0.5433 (3)	0.88069 (19)	0.0313 (7)	
H1	0.1330	0.5104	0.8695	0.038*	
C4	−0.1141 (2)	0.6393 (3)	0.91695 (18)	0.0283 (6)	
C10	−0.2452 (3)	0.3186 (3)	0.74108 (16)	0.0256 (6)	
H10	−0.2342	0.2517	0.7049	0.031*	
C3	−0.0296 (3)	0.6967 (4)	0.9512 (2)	0.0370 (8)	
H3	−0.0362	0.7674	0.9857	0.044*	
C2	0.0618 (3)	0.6478 (4)	0.9335 (2)	0.0381 (8)	
H2	0.1179	0.6839	0.9564	0.046*	
C6	−0.2909 (2)	0.6262 (4)	0.89896 (18)	0.0313 (7)	
H6	−0.3541	0.6573	0.9103	0.038*	
C5	−0.2122 (3)	0.6839 (3)	0.93295 (19)	0.0315 (7)	
H5	−0.2220	0.7540	0.9674	0.038*	

### Atomic displacement parameters (Å^2^)

**Table:**

	*U*^11^	*U*^22^	*U*^33^	*U*^12^	*U*^13^	*U*^23^
Cd	0.01802 (15)	0.01969 (16)	0.02076 (15)	0.000	0.00092 (10)	0.000
S1	0.0382 (4)	0.0350 (4)	0.0306 (4)	−0.0064 (4)	−0.0062 (3)	−0.0011 (3)
N1	0.0205 (11)	0.0264 (12)	0.0242 (11)	−0.0014 (10)	0.0004 (9)	−0.0025 (10)
N2	0.0199 (11)	0.0222 (12)	0.0219 (10)	−0.0003 (10)	0.0006 (9)	0.0008 (9)
N3	0.0250 (14)	0.0317 (15)	0.0356 (14)	0.0000 (11)	−0.0032 (11)	−0.0054 (12)
C13	0.0219 (13)	0.0227 (14)	0.0226 (12)	0.0036 (11)	0.0020 (11)	0.0025 (11)
C12	0.0250 (14)	0.0226 (14)	0.0202 (12)	0.0007 (11)	0.0031 (11)	0.0001 (10)
C9	0.0201 (14)	0.0333 (16)	0.0316 (15)	0.0009 (12)	−0.0022 (12)	0.0022 (12)
C11	0.0219 (13)	0.0212 (13)	0.0215 (12)	0.0014 (11)	0.0027 (10)	0.0031 (10)
C7	0.0255 (14)	0.0263 (15)	0.0245 (13)	0.0032 (12)	0.0043 (11)	0.0030 (11)
C8	0.0213 (14)	0.0360 (17)	0.0338 (15)	0.0067 (13)	0.0012 (12)	0.0033 (13)
C1	0.0267 (15)	0.0338 (17)	0.0333 (15)	−0.0048 (13)	−0.0019 (13)	−0.0078 (14)
C4	0.0304 (16)	0.0263 (15)	0.0282 (14)	0.0002 (13)	0.0034 (12)	−0.0042 (12)
C10	0.0217 (14)	0.0267 (15)	0.0284 (14)	0.0004 (11)	−0.0005 (11)	−0.0015 (11)
C3	0.0374 (18)	0.0364 (19)	0.0371 (18)	−0.0039 (15)	0.0022 (15)	−0.0161 (15)
C2	0.0328 (18)	0.042 (2)	0.0391 (18)	−0.0073 (16)	−0.0018 (15)	−0.0142 (16)
C6	0.0302 (16)	0.0330 (17)	0.0308 (16)	0.0081 (14)	0.0085 (13)	0.0008 (13)
C5	0.0373 (18)	0.0292 (17)	0.0279 (15)	0.0038 (14)	0.0072 (13)	−0.0045 (13)

### Geometric parameters (Å, °)

**Table:**

Cd—N3	2.262 (3)	C9—H9	0.9300
Cd—N3^i^	2.262 (3)	C11—C7	1.411 (4)
Cd—N2^i^	2.369 (3)	C7—C8	1.409 (5)
Cd—N2	2.369 (3)	C7—C6	1.429 (5)
Cd—N1^i^	2.372 (3)	C8—H8	0.9300
Cd—N1	2.372 (3)	C1—C2	1.394 (5)
S1—C13	1.634 (3)	C1—H1	0.9300
N1—C1	1.323 (4)	C4—C3	1.410 (5)
N1—C12	1.365 (4)	C4—C5	1.428 (5)
N2—C10	1.329 (4)	C10—H10	0.9300
N2—C11	1.358 (4)	C3—C2	1.364 (5)
N3—C13	1.135 (4)	C3—H3	0.9300
C12—C4	1.405 (4)	C2—H2	0.9300
C12—C11	1.448 (4)	C6—C5	1.347 (5)
C9—C8	1.362 (5)	C6—H6	0.9300
C9—C10	1.396 (5)	C5—H5	0.9300
			
N3—Cd—N3^i^	95.97 (15)	N2—C11—C12	118.8 (3)
N3—Cd—N2^i^	108.26 (9)	C7—C11—C12	119.3 (3)
N3^i^—Cd—N2^i^	89.42 (10)	C8—C7—C11	117.5 (3)
N3—Cd—N2	89.42 (10)	C8—C7—C6	123.1 (3)
N3^i^—Cd—N2	108.26 (9)	C11—C7—C6	119.4 (3)
N2^i^—Cd—N2	153.81 (12)	C9—C8—C7	120.1 (3)
N3—Cd—N1^i^	90.14 (10)	C9—C8—H8	120.0
N3^i^—Cd—N1^i^	160.29 (9)	C7—C8—H8	120.0
N2^i^—Cd—N1^i^	70.87 (8)	N1—C1—C2	123.1 (3)
N2—Cd—N1^i^	90.48 (8)	N1—C1—H1	118.4
N3—Cd—N1	160.29 (9)	C2—C1—H1	118.4
N3^i^—Cd—N1	90.14 (10)	C12—C4—C3	117.3 (3)
N2^i^—Cd—N1	90.48 (8)	C12—C4—C5	119.7 (3)
N2—Cd—N1	70.87 (8)	C3—C4—C5	123.0 (3)
N1^i^—Cd—N1	90.34 (13)	N2—C10—C9	123.3 (3)
C1—N1—C12	118.2 (3)	N2—C10—H10	118.3
C1—N1—Cd	125.9 (2)	C9—C10—H10	118.3
C12—N1—Cd	115.94 (18)	C2—C3—C4	119.6 (3)
C10—N2—C11	118.5 (3)	C2—C3—H3	120.2
C10—N2—Cd	125.4 (2)	C4—C3—H3	120.2
C11—N2—Cd	116.04 (19)	C3—C2—C1	119.3 (3)
C13—N3—Cd	162.9 (3)	C3—C2—H2	120.4
N3—C13—S1	178.6 (3)	C1—C2—H2	120.4
N1—C12—C4	122.5 (3)	C5—C6—C7	121.2 (3)
N1—C12—C11	118.3 (2)	C5—C6—H6	119.4
C4—C12—C11	119.2 (3)	C7—C6—H6	119.4
C8—C9—C10	118.7 (3)	C6—C5—C4	121.1 (3)
C8—C9—H9	120.7	C6—C5—H5	119.5
C10—C9—H9	120.7	C4—C5—H5	119.5
N2—C11—C7	121.9 (3)		

Symmetry codes: (i) −*x*, *y*, −*z*+3/2.

### Hydrogen-bond geometry (Å, °)

**Table:**

*D*—H···*A*	*D*—H	H···*A*	*D*···*A*	*D*—H···*A*
C8—H8···N3^ii^	0.93	2.54	3.373 (4)	149

Symmetry codes: (ii) *x*−1/2, *y*+1/2, −*z*+3/2.
